# Kidney injury molecule-1 as a predicting factor for inflamed kidney, diabetic and diabetic nephropathy Egyptian patients

**DOI:** 10.1186/s40200-015-0131-8

**Published:** 2015-02-25

**Authors:** Samia A Ahmed, Manal A Hamed

**Affiliations:** Therapeutic Chemistry Department, National Research Centre, 33 El-Bohouth St., ID: 60014618 Dokki Cairo, Egypt

**Keywords:** Kidney injury molecule-1, Diabetes, Biomarkers, Diabetic nephropathy

## Abstract

**Background:**

Kidney injury molecule-1 (KIM-1), a recently discovered transmembrane protein, is expressed in dedifferentiated proximal renal tubular epithelial cells in damaged regions. Kidney injury early detection in diabetic patients has great importance for therapy and prognosis. Therefore, the aim of the present study is to predict, validate and evaluate the presence of KIM-1 in kidney inflammation, dialectic and diabetic nephropathy diseases.

**Methods:**

Sixty males and females subjects (30-52 years) were selected for this study. They were subdivided into three main groups; kidney injury, diabetic and diabetic nephropathy patients. The work was extended to evaluate KIM-1 after treatment of each disease.

**Results:**

The results revealed significant elevation of KIM-1 in the diseased groups and a noticeable reduction after treatment. Diabetic nephropathy recorded the highest KIM-1 level than the AKI state or the diabetic patients. We noticed an association between KIM-1 and sex and a positive correlation (*p <* 0.0001) with the disease severity.

**Conclusions:**

In conclusion, urinary KIM-1 has been reported to be a noninvasive, rapid, sensitive, and reproducible biomarker to detect early kidney injury. We speculate that KIM-1 is expected to be a therapeutic target for kidney injury.

## Introduction

Renal epithelial cell injury is the feature of many acute and chronic renal diseases. Kidney injury molecule-1 (KIM-1), a recently discovered transmembrane tubular protein, is markedly induced in renal injury including acute kidney injury (AKI) and chronic kidney disease (CKD) [1-4]. In renal patients, KIM-1 is up-regulated in a variety of conditions including ischemia, nephrotoxic drugs, CKD, and acute/chronic renal transplant dysfunction. Many studies indicate that KIM-1 is a sensitive and specific marker of kidney injury as well as a predictor of prognosis [5,6]. There are many characteristics of KIM-1 making it an ideal biomarker for kidney injury. For example, KIM-1 is not expressed in normal kidney but specifically expressed in injured proximal tubular cells, and such an expression can persist until the damaged cells have completely recovered. Moreover, the rapid and integrated cleavage of its ectodomain into the lumens of kidney tubules can make it detectable in urine [7]. Urinary KIM-1 level is closely related to tissue KIM-1 and correlates with the severity of renal damage, so quantitation of urinary KIM-1 is likely to be a noninvasive and sensitive method for the evaluation of kidney injury and even for monitoring the therapeutic effects of kidney injury [8].

A long duration of elevated blood glucose levels (i.e. poorly controlled diabetes) is considered the major underlying mechanism causing diabetic nephropathy and chronic kidney disease [9]. Interestingly, the interplay between a disturbed glucose metabolism and kidney damage appears to be bi-directional; individuals with micro-albuminuria are at an increased risk of developing diabetes [10]. Other aspects of glucometabolic derangements than diabetes have also been suggested to be of im portance for the development of kidney damage. In particular, insulin resistance has been shown to be closely associated with the two major indices of kidney damage and dysfunction used in clinical practice, glomerular filtration rate (GFR) and urinary albumin/creatinine ratio (ACR), even prior to the development of diabetes [11]. Both GFR and ACR have limitations as biomarkers as they both mainly reflect an underlying disease process that already is well established [7,12]. In order to better identify individuals with an increased risk for chronic kidney disease, there is need for biomarkers that may detect early signs of kidney damage. A recent study reported elevated KIM-1 levels in diabetes patients with normo-albuminuria [13], indicating that renal tubular damage may be involved in early stages of the development of diabetic nephropathy.

The aim of the present study is to predict and evaluate the KIM-1 level in patients with kidney inflammation, early stage of diabetic nephropathy and diabetic patients with detectable kidney inflammation. In addition, the role of KIM-1 to be consider as a diagnostic tool after treatment of such disease.

## Materials and methods

### Subjects

Participants were recruited from Kasr Al-Aini Clinics at Kasr Al-Aini university hospital, Cairo, Egypt. This research was performed on 60 males and 60 females aged 30-52 years. Each sex was classified into four main groups (15 subjects each). Group 1 served as healthy control. Group 2 was patients with kidney inflammation (KI). Subgroup 2 was the same patients of group 2 and undergoes the classical treatment of inflamed kidney. Group 3 was diabetic patients (insulin independent). Subgroup 3 was the same members of group 3 and treated for diabetes. Group 4 was patients with diabetic nephropathy. Subgroup 4 was diabetic nephropathy patients of group 4 and treated for diabetes and kidney inflammation.

### Clinical examination and diagnosis

Serum and urine samples were collected from healthy subjects who attending to Kasr Al-Aini Clinics, Cairo, Egypt for regular checkup. Individuals were informed of the testing to be performed and only the specified testing was performed on the collected reference specimens.

Analyses were conducted with the full respect for the individual’s right to confidentiality. Information regarding their age and gender was also collected to estimate the glomerular filtration rate (GFR), urine and serum creatinine as well as blood glucose level in all the studied groups.

KIM-1 patients, diabetic and diabetic nephropathy patients were diagnosed according to the reference values, where diabetes was defined as fasting plasma glucose ≥7.0 mmol/l (≥126 mg/dl) [14], glomerular filteration rate (GFR) <60 mL/min/1.73 m2 [15] and serum creatinine level > 0.5 mg/dL [1].

### Treatments

AKI patients were treated with 500 mg of Cpirofloxacin (Amriya Pharm Industries Co., Egypt) twice daily for 7 days and Urosolvine sachets (Nile Pharm Co., Egypt) three times daily. Diabetic patients treated with 1 g of Glucophage (Mina Pharm Co., Egypt) tablets daily. Diabetic nephropathy patients treated with the three above drugs for 7 days before the biochemical determinations of KIM-1.

### Sample collections for KIM-1 determination

Urine samples were allowed to sit at room temperature for 30 minutes to sediment, and the supernatant was aliquoted and stored at -80°C until analysis.

### KIM-1 determination

Construction of KIM-1 sandwich ELISA (R&D Cat#DY1750, Minneapolis, MN): The wells of Nunc-Maxisorp EIA plates were coated by diluting the capture antibody (72 μg/mL) to a working concentration of 0.4 μg/mL in PBS with 100 μL in each well. The plate was sealed and incubated overnight at room temperature. Each well was aspirated and washed using an automated microplate washer (Bio-Tek) with 400 μL of Wash Buffer (0.05% Tween-20 in PBS), repeating the process two times for a total of three washes. The plates were blocked by adding 300 μL of reagent diluent (1% BSA in PBS, 0.2 μm filtered) to each well and incubated at room temperature for 2 hours. After washing as in the previous step, 100 μL of standard recombinant human KIM-1 (0-2000 pg/mL), control, and urine sample was pipette to the designated well, covered with an adhesive strip, and placed on the orbital shaker at 400 rpm at room temperature for 2 hours. The plate was washed using the same wash protocol as before, and 100 μL of the biotinylated goat anti-human KIM-1 detection antibody diluted in reagent diluent to a working concentration of 400 ng/mL was added to each well. The plate was covered with a new adhesive strip, incubated at room temperature for 2 hours with continued shaking at 400 rpm. Washing step was repeated and 100 μL of streptavidin-HRP diluted to a working dilution was added to each well. The plate was protected from light, covered with a new adhesive strip, shaken at 400 rpm, and incubated at room temperature for 20 minutes. After washing, 100 μL substrate solution was added to all wells, protected from light, covered with an adhesive strip, shaken at 400 rpm, and incubated at room temperature for 7 minutes. The reaction was stopped by adding 50 μL of stop solution to all wells. The absorbance was measured using a plate reader (BioTek Elx800) at 450 nm with an absorbance correction at 540 nm. The urinary KIM-1 concentration was calculated based on the standard curve and expressed in absolute terms (pg/mL).

### Statistical analysis

Results were expressed as mean ± SD of 15 subjects in each group. Results were evaluated by using one way analysis of variance test (ANOVA), Costat Software Computer Program with least significance difference (LSD) between groups at *p <* 0.05. Pearson’s test was done to detect correlation value with each parameter.

### Human rights

All procedures followed were in accordance with the ethical standards of the Medical Ethical Committee at National Research Centre, Cairo, Egypt and with the Helsinki Declaration of 1975, as revised in 2008.

### Informed consent

Informed consent was obtained from all patients for being included in this study.

## Results and discussion

**Figure 1:**
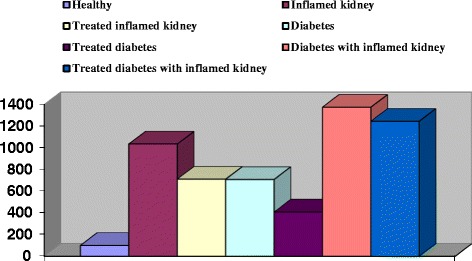
**% changes of KIM-1 in males patients with inflamed kidney, diabetes and inflamed kidney with diabetes.**

**Figure 2:**
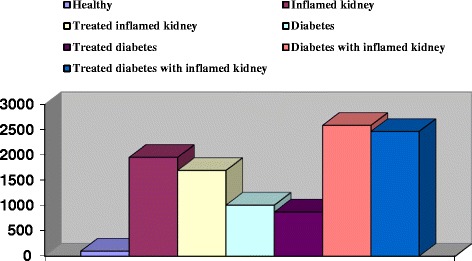
**KIM-1 in females patients with inflamed kidney, diabetes and inflamed kidney with diabetes.**

The ability of biomarkers to predict kidney inflammation has been studied intensely in several different clinical settings. For a sound interpretation of the reported results, it is important to realize that the studies present a mixture of ‘AKI diagnosis confirmation’ in patients with established AKI and ‘AKI early prediction’ in patients with developing AKI. AKI has been defined as a rapid decline in glomerular filtration rate that occurs over hours or days [7]. For the clinical application of a new biomarker, it should prove to be more accurate with earlier detectable than the current gold standard serum creatinine, which implies ‘early prediction’ only [16]. The present results revealed significant increase in KIM-1 by 935.70 and 1854.05% in early detected inflamed kidney of males and females as compared with the control group (Figures 1 and 2). Although the KIM-1 basal line was higher in males 280.38 ± 51.66 pg/ml than female 147.18 ± 21.70, but it was highly detectable in females inflamed kidney than males (Tables 1 and 2). Inflamed kidney patient that undergo treatment recorded significant decrease in KIM-1by 31.25% in males and 30.54% in females as compared to the inflamed kidney patients. Therefore, KIM-1 recorded efficiency in diminution after treatment by 323.59 and 259.20% in males and females, respectively (Tables 1 and 2). We also noticed insignificant correlations between KIM-1and age, while a highly association between KIM-1 and sex was observed regarding to its high expression in healthy male. Highly positive association between KIM-1 and degree of kidney inflammation was also observed. Ruangyuttikarn et al. [17] had also proved that detection of KIM-1 by ELISA technique gave a very high sensitivity and specificity in urine samples. In addition, KIM-1 in urine was still stable even after the urine was frozen and thawed for 4 cycles. The same authors added that KIM-1 more accurate for detection of kidney inflammation that the two conventional renal tubular dysfunction biomarkers; N-acetyl-β-Dglucosaminidase (NAG) and β2-microglobulin (β2-MG).

**Table 1:** **Kidney inflammatory molecule-1 in inflamed kidney, diabetes and inflamed kidney with diabetes in males patients**

**Groups**	**Mean ± SD**	**% of KIM-1 efficiency**	**Correlation between KIM-1 and disease severity**	
			**r**	**P<**
Healthy	280.38^f^ ± 51.66	---	---	---
Inflamed kidney	2903.09^c^ ± 301.76	---	1.00^*^	0.0001
Treated inflamed kidney	1995.80^d^ ± 80.87 [-31.25]	323.59	0.86^*^	0.0010
Diabetes	1987.78^d^ ± 169.35	---	0.72^*^	0.0200
Treated diabetes	1146.56^e^ ± 46.35 [-42.31]	300.02	0.75	0.0800
Diabetes with inflamed kidney	3855.80^a^ ± 116.95	---	0.94^*^	0.0001
Treated diabetes with inflamed kidney	3491.26^b^ ± 112.39 [-9.45]	130.02	0.83^*^	0.0010

Data are expressed as mean ± SD of 15 males in each group.

Values are expressed as pg/ml.

Statistical analysis is carried out using one way analysis of variance test (ANOVA), Costat Computer Program accompanied with least significance difference between groups (LSD) at *p <* 0.05.

Unshared superscript letters between groups are the significance value at *p <* 0.0001.

*is the significance level between the KIM-1 level and the disease severity.

Values between parentheses are percentage change over diseased group.

% of efficiency = [(mean of treated group- mean of diseased group)/mean of healthy group] × 100.

**Table 2:** **Kidney inflammatory molecule-1 in inflamed kidney, diabetes and inflamed kidney with diabetes in females patients**

**Groups**	**Mean ± SD**	**% of KIM-1 efficiency**	**Correlation between KIM-1 and disease severity**	
			**r**	**P<**
Healthy	147.18^e^ ± 21.70	---	---	---
Inflamed kidney	2875.98^b^ ± 275.72	---	0.86^*^	0.0001
Treated inflamed kidney	2497.43^c^ ± 254.22 [-30.54]	257.20	0.76^*^	0.0300
Diabetes	1485.81^d^ ± 18.50	---	0.72	0.0080
Treated diabetes	1288.72^d^ ± 36.54 [-13.16]	133.91	0.94^*^	0.0010
Diabetes with inflamed kidney	3806.52^a^ ± 108.19	---	0.81^*^	0.0001
Treated diabetes with inflamed kidney	2630.16^b^ ± 91.08 [-30.90]	119.82	0.87^*^	0.0001

Data are expressed as mean ± SD of 15 females in each group.

Values are expressed as pg/ml.

Statistical analysis is carried out using one way analysis of variance test (ANOVA), Costat Computer Program accompanied with least significance difference between groups (LSD) at *p <* 0.05.

Unshared superscript letters between groups are the significance value at *p <* 0.0001.

*is the significance level between the KIM-1 level and the disease severity.

Values between parentheses are percentage change over diseased group.

% of efficiency = [(mean of treated group- mean of diseased group)/mean of healthy group] × 100.

Regarding to the diabetic patients in our results, we noticed a significant increase in KIM-1in both males and females, although these patients not recorded any sign of kidney inflammation. The KIM-1 in male diabetic patient was significantly increase by 608.95% (Figure 1), while it was significantly increase by 909.51% in female diabetic patients (Figure 2). In agreement with our results, Chaudhary et al. [12] and Rossing [18] stated that early renal tubular damage biomarker levels (including urinary KIM-1 levels) are elevated in patients with diabetes, even in those with normoalbuminuria. Carlsson et al. [15] added that insulin sensitivity was inversely and independently associated with urinary KIM-1 concentrations.

Patients undergo classical diabetic treatment recorded significant decrease in KIM-1 by 42.31% in male and insignificant decrease in female by 13.16% as compared with the diabetic persons. Therefore, KIM-1 was improved after treatment by 300.02% in male and 133.91% in female and correlate positively with the high glucose level (Tables 1 and 2).

Concerning with diabetic nephropathy patients, the present study revealed significant increase in KIM-1 in males and females by 1275.20 and 2486.30%, respectively as compared with the healthy group (Figures 1 and 2). We noticed that KIM-1 level was more pronounced in females of diabetic nephropathy than in males. Positive correlations between KIM-1 level and both sex and severity of the disease were also recorded. In parallel with our finding Carlsson et al. [15] found significant elevation of KIM-1 in insulin resistance elderly individuals. Impaired insulin sensitivity and compensatory hyperinsuli- nemia have been suggested to contribute to development of renal injury by promotion of mitogenic and fibrotic processes via different pathophysiologic pathways such as activation of insulin-like growth factor-1, transforming growth factor-b, endothelin-1, and the renin–angiotensin–aldosterone system [19]. Moreover, insulin resistance is closely associated with oxidative stress [20], pro-inflammatory cytokines and adipo-kines [21], which also could promote renal injury. But the opposite chain of events is also possible; an increased inflammatory activity due to ongoing kidney damage could also impair insulin sensitivity [22]. Tubulointer- stitial injury is present in all forms of chronic kidney disease and is thought to be a better predictor of disease progression and long-term prognosis than is the severity of damage to glomeruli [23]. By measuring KIM-1, this “tubular phase” of renal damage could be detected before the development of albuminuria, the currently used marker of early diabetic nephropathy [18,19].

We noticed that the KIM-1 in diabetic nephropathy patients recorded the highest level than in kidney inflammation state alone. This is may be due to the severity state of kidney inflammation associated with the diabetic state. Diabetic patients only may not have a complication as kidney inflammation or it may not reached to the diagnosis level which give an additional support to use KIM-1 as a diagnostic tool.

In the present study, treatment of diabetic nephropathy patients showed significant decrease in KIM-1 level by 9.45% and 30.90% in male and females, respectively as compared with the diabetic nephropathy groups (Tables 1 and 2). Therefore, KIM-1 recorded efficiency in diminution after treatment by 130.02% and 119.82% in males and females, respectively. Vaidya et al. [24] mentioned that regression of diabetic nephropathy after treatment has been shown to be associated with reduced levels of urinary KIM-1 which confirmed our results.

In conclusion, KIM-1 might be a specific predictor for early detection of kidney inflammation, in diabetic disease where no sign of kidney inflammation was persisted, and in diabetic nephropathy disease. KIM-1 is expected to be a therapeutic target for kidney injury. KIM-1 and its potential value need to be validated in large studies and across a broader scope of clinical settings. Due to the relatively limited number of cases in this study, further studies are needed for accurately determine the use of KIM-1 as a biomarker, as the small sample size makes it difficult to evaluate its validity.
